# An investigation into the molecular basis of cancer comorbidities in coronavirus infection

**DOI:** 10.1002/2211-5463.12984

**Published:** 2020-10-12

**Authors:** Antonio Facchiano, Francesco Facchiano, Angelo Facchiano

**Affiliations:** ^1^ Istituto Dermopatico dell'Immacolata IDI‐IRCCS Rome Italy; ^2^ Department of Oncology and Molecular Medicine Istituto Superiore di Sanità Rome Italy; ^3^ Institute of Food Science National Research Council Avellino Italy

**Keywords:** comorbidity, coronavirus, kidney cancer, prostate cancer, skin cancer, thyroid cancer

## Abstract

Comorbidities in COVID‐19 patients often worsen clinical conditions and may represent death predictors. Here, the expression of five genes, known to encode coronavirus receptors/interactors (*ACE2*, *TMPRSS2*, *CLEC4M*, *DPP4* and *TMPRSS11D*), was investigated in normal and cancer tissues, and their molecular relationships with clinical comorbidities were investigated. Using expression data from GENT2 databases, we evaluated gene expression in all anatomical districts from 32 normal tissues in 3902 individuals. Functional relationships with body districts were analyzed by chilibot. We performed DisGeNet, genemania and DAVID analyses to identify human diseases associated with these genes. Transcriptomic expression levels were then analyzed in 31 cancer types and healthy controls from approximately 43 000 individuals, using GEPIA2 and GENT2 databases. By performing receiver operating characteristic analysis, the area under the curve (AUC) was used to discriminate healthy from cancer patients. Coronavirus receptors were found to be expressed in several body districts. Moreover, the five genes were found to associate with acute respiratory syndrome, diabetes, cardiovascular diseases and cancer (i.e. the most frequent COVID‐19 comorbidities). Their expression levels were found to be significantly altered in cancer types, including colon, kidney, liver, testis, thyroid and skin cancers (*P* < 0.0001); AUC > 0.80 suggests that TMPRSS2, CLEC4M and DPP4 are relevant markers of kidney, liver, and thyroid cancer, respectively. The five coronavirus receptors are related to all main COVID‐19 comorbidities and three show significantly different expression in cancer versus control tissues. Further investigation into their role may help in monitoring other comorbidities, as well as for follow‐up of patients who have recovered from SARS‐CoV‐2 infection.

## Graphical abstract

Five receptors/interactors of coronaviruses may play a key role in COVID‐19 comorbidities. They are expressed in almost any body district and, regardless of coronavirus infection, are associated with several diseases, including those most frequently cooccurring in COVID‐19 patients. Their expression is strongly altered in many cancers and at least three of them may be relevant markers in kidney, liver and thyroid cancers.

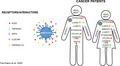

**Abbreviations**
ACE2: angiotensin converting enzyme 2
AUC: area under the curve
CLEC4M: C‐type lectin domain family 4 member M
DPP4: dipeptidyl peptidase 4
HCV: hepatitis C virus
ROC: receiver operating characteristic
TMPRSS11D: transmembrane protease serine 11D
TMPRSS2: transmembrane protease serine 2

The COVID‐19 pandemic is now affecting almost all countries. Patients with severe clinical conditions are often affected by other pathologies, the most frequent being diabetes, coronary heart diseases, cerebrovascular diseases [1] and cancer [2]. SARS‐CoV‐2 virus directly affects mainly the lung tissues and the higher respiratory tract. Nevertheless, besides the lungs and lung fluids, SARS‐CoV‐2 has been found in body districts such as feces (approximately 30% of cases) and in the blood (1% of cases) [3]. Furthermore, acute kidney injury, proteinuria and hematuria were found to be associated with the death of COVID‐19 patients [4] and other organs, such as the intestine, testis and kidney, have been proposed as possible transmission routes [5]. Previous studies have demonstrated the large diffusion of other coronavirus strains (i.e. SARS‐CoV) throughout almost the entire body [6]. In the present study, we focused on the molecular bases possibly underlying the comorbidities observed in SARS‐CoV‐2 infection. We investigated the genes involved as receptors or main interactors of SARS‐CoV‐2 and similar coronaviruses responsible for SARS and MERS. Specifically, ACE2, TMPRSS2, CLEC4M, DPP4 and transmembrane protease serine 11D (TMPRSS11D) were analyzed by assessing their RNA expression levels in different body districts and in different cancer types. Angiotensin converting enzyme 2 (ACE2) is a carboxypeptidase that converts angiotensin I to angiotensin 1–9 and angiotensin II to angiotensin 1–7. It is recognized as the receptor of SARS‐CoV and SARS‐CoV‐2 viruses [7]. Transmembrane protease serine 2 (TMPRSS2) is a serine protease up‐regulated by androgen hormones; it is involved in the infection process of many viruses, including coronaviruses, acting on the spike proteins and on ACE2, facilitating virus‐cell membrane fusion [8, 9]. CLEC4M, DPP4 and TNPRSS11D are reported to be receptors or interactors of other coronaviruses. Although their receptor‐activity for SARS‐CoV‐2 has not been demonstrated to date, numerous evidence is available demonstrating their role in related coronaviruses. C‐type lectin domain family 4 member M (CLEC4M) is a membrane protein involved as an attachment site of many viruses, including SARS; it is a receptor with pathogen recognition capability toward several parasites and viruses and has cell adhesion properties. It is a known attachment receptor for Ebola virus, hepatitis C virus (HCV), human coronavirus 229E and SARS coronavirus, amongst others [10]. Dipeptidyl peptidase 4 (DDP4) is a serine exopeptidase, corresponding to the T‐cell activation antigen CD26. It is a glycoprotein membrane receptor involved in T‐cell activation, with peptidase enzymatic activity. It is known as the MERS receptor [11]. TMPRSS11D is a serine protease, active on ACE2 as well as on viral spike proteins. It cleaves and activates the spike glycoprotein of human coronavirus 229E (HCoV‐229E), facilitating its cell entrance [12, 13]. Such molecules are strongly involved in several biological functions and in the present study their associations with human diseases, as well as their transcription expression levels, were assessed in 31 different human cancer types compared to healthy controls, from approximately 43 000 individuals.

## Results

### Expression level of coronavirus receptors/interactors in normal tissues

The transcriptomic expression levels of the five receptors/interactors of SARS‐CoV‐2 and other human coronaviruses were investigated in the human body districts. Specifically, ACE2, TMPRSS2, CLEC4M, DPP4 and TMPRSS11D expression levels in normal tissues were derived from the GENT2 database (http://gent2.appex.kr/gent2), containing data from about 28 000 controls and cancer subjects (angiotensin converting enzyme 2; for further details on tissues and numerosity, see Table S1). Interestingly, the five coronaviruses receptors were found to be expressed in almost any anatomical district. Expression in normal tissues was analyzed in more detail compared to differential expression in cancer types, as reported below. Ubiquitous expression was confirmed by an additional analysis carried out via the Human Protein Atlas (https://www.proteinatlas.org/), showing both protein and RNA expression in all body districts (Fig. S1). The almost ubiquitous expression of coronavirus receptors led us to hypothesize that their biological action may affect many organs and tissues. To investigate this hypothesis, a chilibot analysis was carried out (http://www.chilibot.net/). The presence of interactive relationships was analyzed by measuring co‐occurrence of the given keywords in the same sentence within the manuscript’s abstract. Table 1 shows that most anatomical districts share interactive relationships with ‘coronavirus’ word. Districts such as colon, liver, testis, lung and kidney show the highest relationships with coronavirus, as reported in Table 1, and also show the highest RNA expression levels in many cases (Fig. S1).

**Table 1:** Functional relationships of coronavirus with anatomic districts. Analysis performed according to chilibot relates to the most relevant and numerous functional interactions reported in PubMed abstracts. Strength of the relationship is measured by the number of supporting references. ‘ ✓✓✓’ corresponds to more than 30 references supporting the relationship; ‘✓✓’ corresponds to between 15 and 30 references supporting the relationship; and ‘✓’ corresponds to less than 15 references supporting the relationship. –, no references. The ‘only interactive relationship’ filter was active.

Body district	Presence of an interactive relationship with the word ‘coronavirus’	Body district	Presence of an interactive relationship with the word ‘coronavirus’
Lung	✓✓✓	Rectum	✓
Brain	✓✓✓	Stomach	✓
Heart	✓✓✓	Thyroid	✓
Liver	✓✓✓	Tonsil	✓
Colon	✓✓✓	Salivary gland	✓
Skin	✓✓✓	Endocrine	✓
Kidney	✓✓✓	Bladder	✓
Small intestine	✓✓✓	Tongue	✓
Blood	✓✓✓	Prostate	✓
Trachea	✓✓✓	Uterus	–
Spleen	✓✓✓	Pleura	–
Lymph node	✓✓✓	Esophagus	–
Testis	✓✓✓	Parathyroid	–
Thymus	✓✓✓	Gallbladder	–
Ovary	✓✓	Placenta	–
Breast	✓✓	Cervix	–
Duodenum	✓✓	Adrenal gland	–
Pancreas	✓		

### Human diseases associated with ACE2, TMPRSS2, CLEC4M, DPP4 and TMPRSS11D

Table 2 highlights diseases associated with ACE2, TMPRSS2, CLEC4M, DPP4 and TMPRSS11D, according to the widely used DisGeNET database (https://www.disgenet.org). Such genes were found to strongly associate with several human diseases, including the most frequently observed COVID‐19 comorbidities, such as severe acute respiratory syndrome, diabetes, and heart and kidney diseases. Interestingly, a few cancer types (namely prostate, breast and ovary cancers), as well as tumor progression, carcinogenesis and metastasis, were found to be associated with these genes. Infections other than coronavirus were found to be associated with these genes, namely influenza, HIV, HCV and trypanosomiasis infections.

**Table 2:** Diseases associated with genes interactors of coronavirus. Strength of association is measured by the number of supporting references, according to the DisGeNET database. ‘✓’ indicates one to nine peer‐reviewed studies reporting the association and ‘✓✓’ indicates 10 or more peer‐reviewed studies reporting the association.

	ACE2	TMPRSS2	CLEC4M	DPP4	TMPRSS11D
Diabetes/obesity/diabetic nephropathy	✓✓			✓✓	
Severe acute respiratory syndrome/middle east respiratory disease	✓✓		✓	✓	
Congestive heart failure/atherosclerosis/aortic valve insufficiency	✓✓	✓		✓	
Prostate cancer		✓✓			✓
Hypertensive disease	✓✓				
Kidney disease	✓✓				
von Willebrand disease, type 1/venous thromboembolism			✓	✓	✓
Carcinogenesis/metastatization/tumor progression		✓	✓	✓	
Infection/influenza/		✓	✓		
Colorectal cancer			✓	✓	
African trypanosomiasis					✓
Ovarian cancer				✓	
Breast cancer					✓
HIV infection			✓		
Hepatitis C			✓		
Familial lichen amyloidosis		✓			

**Fig. 1:**
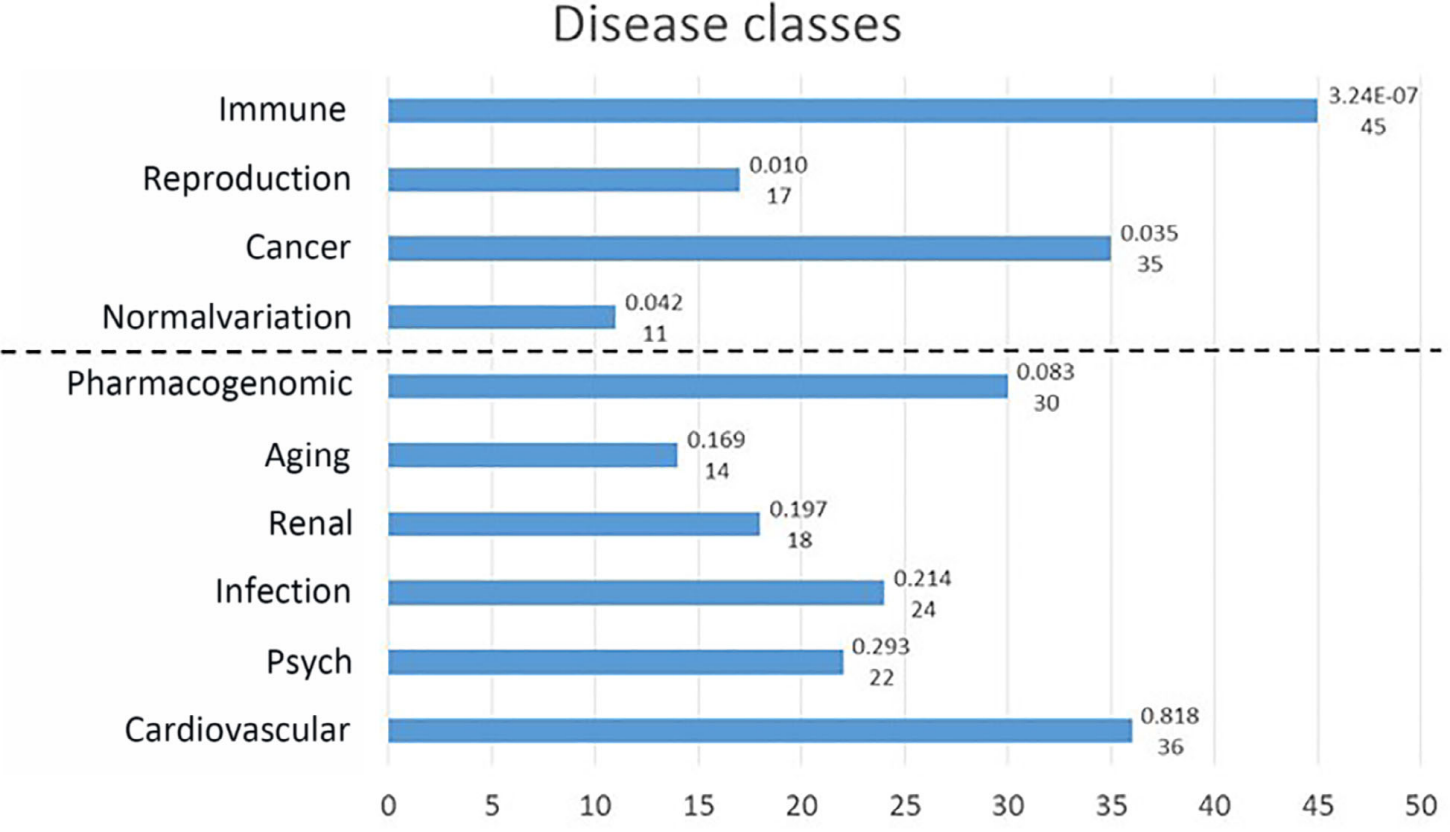
The GAD disease classes associated with the overall list of 105 genes. Numbers on the right indicate the *P*‐value (Bonferroni Šidák *P*‐value according to the DAVID manual) and the number of genes involved, according to DAVID analysis. The dash line separates classes with a significance below the threshold (*P* < 0.05: ‘Immune’, ‘Reproduction’, ‘Cancer’ and ‘Normalvariation’) from classes with a significance above the threshold. ‘Normalvariation’ is the name of one of the 18 sets of genes associated with diseases in genome‐wide association studies and other genetic association datasets from the GAD High Level Gene‐Disease Associations dataset. More details about diseases classes and genes included are available at the GAD link: https://maayanlab.cloud/Harmonizome/dataset/GAD+High+Level+Gene‐Disease+Associations.

As a further investigation, we used a gene‐enrichment approach by selecting, for each of the five genes, a list of the 20 most related genes. The five lists were combined and investigated with the DAVID (https://david.ncifcrf.gov) and Genetic Association Database (https://maayanlab.cloud/Harmonizome/dataset/GAD+High+Level+Gene-Disease+Associations) to detect gene–disease relationships. The combination of the five genes ACE2, TMPRSS2, CLEC4M, DPP4 and TMPRSS11D with the 20 genes most related to each of these genes (i.e. 5 + 100) shows a significant relationship with the diseases classes as reported in Fig. 1. The disease class showing the best association is ‘IMMUNE’, related to 45 genes in the list, with high significance (*P* = 3.24 × 10^–7^). Relevant associations were also found with ‘REPRODUCTION’, ‘AGING’ and ‘CANCER’ disease classes.

The analysis has been also performed for the five separate lists, and the results confirm the evidence from the DisGenNET analysis (a complete list of diseases associated with the five separate lists os provided in Table S2).

### Expression of ACE2, TMPRSS2, CLEC4M, DPP4 and TMPRSS11D in 31 human cancer types

**Fig. 2:**
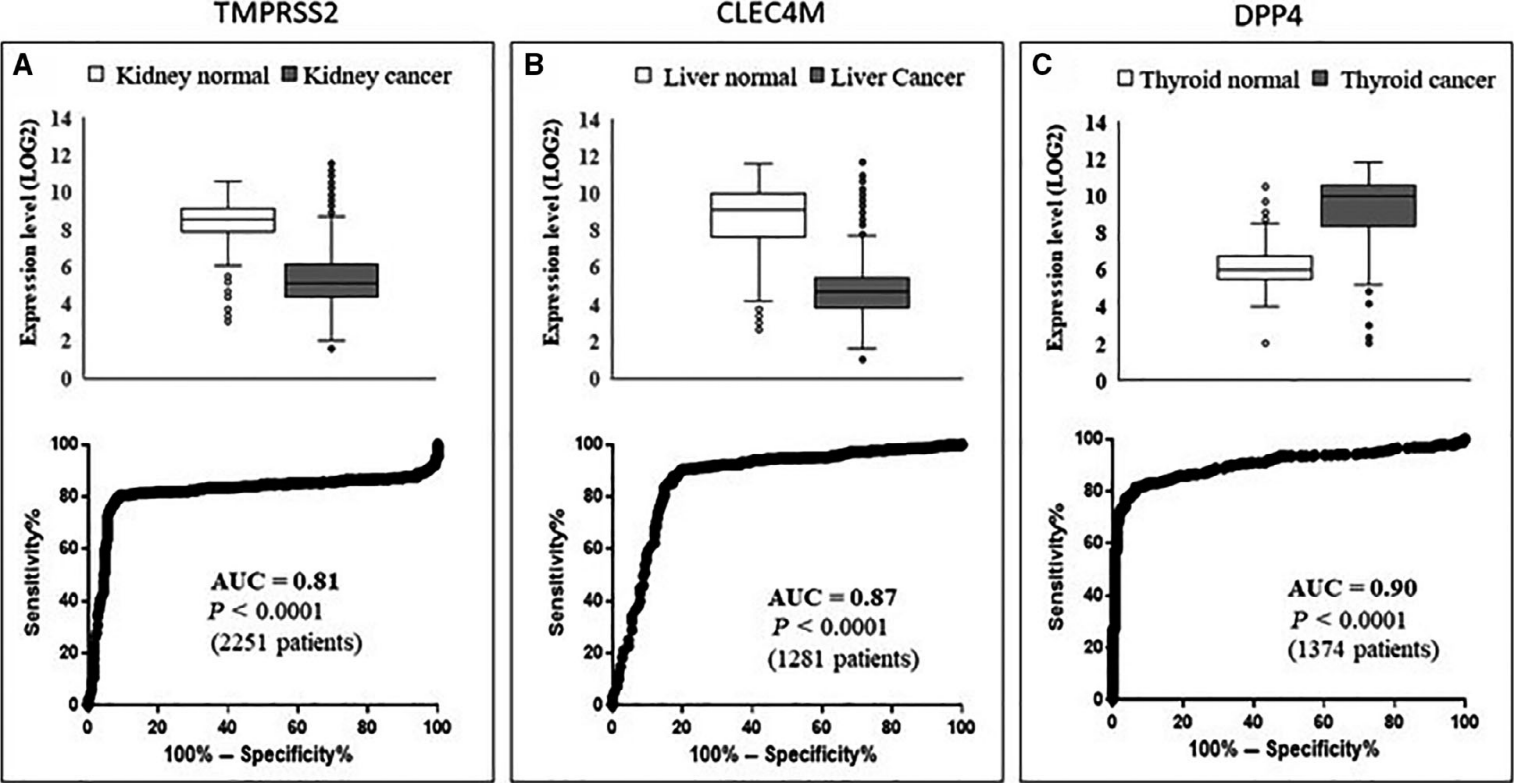
Differential expression in cancer versus normal tissues. Top: Differential RNA expression in cancer versus controls for TMPRSS2 (A), CLEC4M (B) and DPP4 (C) retrieved from the GENT2 database in kidney, liver and thyroid cancers, respectively. Bottom: ROC analysis carried out on data corresponding to the top panels. Boxes are obtained using excel and report the median value, quartiles and outliers.

**Fig. 3:**
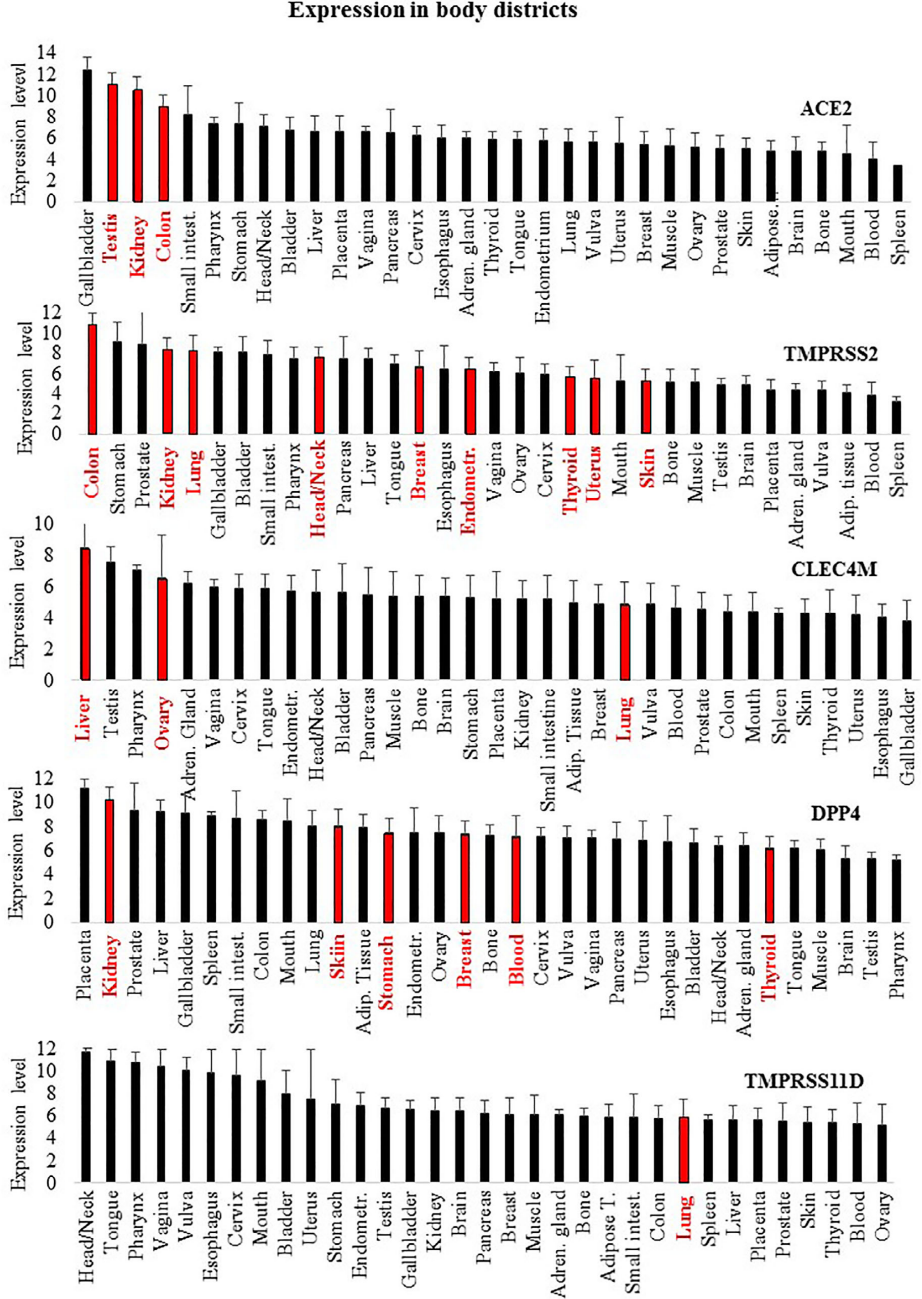
Sites of normal expression are reported, sorted from the highest to the lowest RNA expression level. Sites where the expression in cancer conditions is significantly changed are highlighted in red. ACE2: three out of the four sites showing the highest expression in normal tissues match with cancer types where ACE2 expression is significantly changed. TMPRSS2: three out of the five sites showing the highest expression in normal tissues match with cancer types where TMPRSS2 expression is significantly changed. CLEC4M: two of the four sites showing the highest expression in normal tissues match with cancer types where CLEC4M expression is significantly changed. DPP4: one of the two sites showing the highest expression in normal tissues match with cancer types where DPP4 expression is significantly changed. Data are expressed as the mean ± SD.

Given the strong relationships found with different cancer types, we then focused our analysis on the expression level of these genes in a large number of different cancer types, by analyzing transcriptomic datasets. Table 3 reports cancer types showing significantly (*P* < 0.0001) different RNA expression compared to appropriate healthy control tissues. The analysis was carried out via the GEPIA2 database (http://gepia2.cancer‐pku.cn/#index), containing RNA expression data from 14 768 patients classified in 31 cancer types and normal controls (further details on cancer types and the number of subjects are provided in Table S1). Validation of such analysis was carried out on an independent database, namely GENT2, containing data from 28 228 patients stratified in 21 cancer types and normal controls (Table S1). Table 3 shows the presence of significant differential expression in cancer versus normal controls in several cases. Namely, ACE2 has validated significant differential expression in colon, kidney, testicular and thyroid cancers; TMPRSS2 has validated significant differential expression in breast, colon, head and neck, kidney, lung, skin and uterus cancers; CLEC4M has validated significant differential expression in liver, lung and ovary cancers; DPP4 has validated significant differential expression in breast, kidney, blood, skin, stomach and thyroid cancers; and TNPRSS11D has validated significant different expression in lung cancer. Figure 2 shows the most relevant differential expression and the corresponding area under the curve (AUC) according to receiver operating characteristic (ROC) analysis of TMPRSS2 (Fig. 2A), CLEC4M (Fig. 2B) and DPP4 (Fig. 2C), respectively. The AUC > 0.80 shown by TMPRSS2, CLEC4M and DPP4 suggests that these genes may act as effective molecular markers for kidney, liver and thyroid cancers. Combining data taken regarding expression levels in normal tissues and from cancer types with a validated differential expression versus the corresponding normal tissues (Table 3) led to an interesting observation, as summarized in Fig. 3: the normal tissues where such genes have the highest expression levels match with cancer types where these genes show a validated differential expression (red columns in Fig. 3). This is evident for ACE2, TMPRSS2, CLEC4M and DPP4, as depicted in Fig. 3.

**Table 3:** Transcriptomic analyses: differential gene expression of coronavirus receptors/interactors in cancer versus normal tissues. ‘✓’ indicates a significant change in cancer versus healthy controls observed in the GEPIA2 database and ‘✓✓’ indicates significant change in cancer versus controls observed in the GEPIA2 database and validated in the GENT2 database. Empty cells indicate no significant difference observed in the GEPIA2 database. Significance threshold: *P* < 0.0001.

Cancer type (GEPIA2 database)	ACE2	TMPRSS2	CLEC4M	DPP4	TMPRSS11D
Adrenocortical carcinoma	–	–	–	–	–
Bladder urothelial carcinoma	–	–	–	–	–
Breast invasive carcinoma	–	✓✓	–	✓✓	–
Cervic squamous cell carcinoma/endocervical adenocarcinoma	–	–	–	–	–
Cholangiocarcinoma	✓	–	✓	–	–
Colon adenocarcinoma	✓✓	✓✓	–	–	–
Lymphoid neoplasm diffuse large B‐cell lymphoma	–	–	–	–	–
Esophageal carcinoma	–	✓	–	✓	✓
Glioblastoma multiforme	–	–	–	–	–
Head and neck squam cell carcinoma	–	✓✓	–	–	–
Kidney chromophobe	✓✓	✓✓	–	✓✓	–
Kidney renal clear cell carcinoma	–	✓✓	–	–	–
Kidney renal papillary cell carcinoma	✓✓	✓✓	–	✓✓	–
Acute myeloid leukemia	–	–	–	✓✓	–
Brain lower grade glioma	–	–	–	–	–
Liver hepatocellular carcinoma	–	–	✓✓	✓	–
Lung adenocarcinoma	–	–	✓✓	✓	–
Lung squamous cell carcinoma	–	✓✓	✓✓	✓	✓✓
Ovarian serous cystadenocarcinoma	–	–	✓✓	–	–
Pancreatic adenocarcinoma	✓	–	–	✓	–
Pheochromocytoma and paraganglioma	–	–	✓	–	–
Prostate adenocarcinoma	–	✓	–	✓	–
Rectum adenocarcinoma	✓	✓	–	–	–
Sarcoma	✓	✓	–	–	–
Skin cutaneous melanoma	–	✓✓	–	✓✓	–
Stomach adenocarcinoma	–	–	–	✓✓	–
Testicular germ cell tumor	✓✓	✓	✓	–	–
Thyroid carcinoma	✓✓	✓	–	✓✓	–
Thymoma	–	–	–	✓	–
Uterine corpus endometrium carcinoma	–	✓✓	–	–	–
Uterine carcinosarcoma	–	✓✓	–	–	–

## Discussion

The present study investigated the hypothesis that specific molecular bases may underlie the observed comorbidities of COVID‐19, specifically involving coronavirus receptors/interactors. Molecular expression analyses and gene–disease association analyses were carried out to investigate the role coronavirus interactors may play in such comorbidities. We followed a methodology based on gene expression analyses and validation, which was previously shown to be an effective approach in cancer markers investigations [14, 15, 16]. Five molecules known to be involved in coronavirus infection were investigated: ACE2, TMPRSS2, CLEC4M, DPP4 and TMPRSS11D.

Additional molecules have been proposed to control virus entry [17]. Figure 3 and Fig. S1 show that the expression of such molecules is not limited to body infection sites; rather, they appear to be almost ubiquitously expressed in all body districts at both RNA and protein levels. This observation parallels the data reported in Table 1 indicating that coronavirus shares interactive relationships with many body districts, including the small intestine, lung, heart, kidney, testis, ovary and breast, where receptors are highly expressed. Furthermore, Fig. 1 and Table 2 report several human diseases and disease classes associated with ACE2, TMPRSS2, CLEC4M, DPP4 and TMPRSS11D. Many diseases reported in Fig. 1 and Table 2 are frequent COVID‐19 comorbidities, namely hypertension, diabetes, cardiovascular diseases, respiratory system disease, kidney diseases and cancers [2, 18, 19], suggesting that their occurrence in COVID‐19 patients may be pathogenetically related to the molecules regulating virus entry. Kidney comorbidity, hypertension and diabetes mellitus have been suggested as death predictors in coronavirus patients [20, 21]. According to the high expression values of ACE2, TMPRSS2, CLEC4M, DDP4 and TMPRSS11D in the normal skin compartment indicated in Fig. 3, as well as the large interactive relationships with skin tissue reported in Table 1, we may hypothesize comorbidity signs in coronavirus patients at the skin level. Indeed, this has been confirmed in a very recent study highlighting dermatological manifestations in approximately 20% of COVID‐19 patients [22]. We focused the present study on cancer comorbidity in COVID‐19 patients, which was recently shown to reach a rate of up to 11% [23]. The Istituto Superiore di Sanità, an institution of the Italian Ministry of Health, has recently reported the percentage of the most common co‐existing diseases in COVID‐19 patients [24], showing that 19.5% of COVID‐19 deceased patients had active cancers in the preceeding 5 years. Ongoing studies are investigating the severity of clinical conditions in COVID‐19 patients with respect to the underlying comorbidities; case fatality rates are under investigation, and almost all COVID‐19‐related deaths (i.e. 94%) occur in patients with underlying conditions [25] and, according to the John Hopkins ABX Guide [26] ‘… people with comorbidities appear more likely to develop an infection and severe symptoms and be at risk for death’. Although the numbers analyzed are now relatively small, observations are being reported regarding the frequency and severity of cancer co‐occurrence in COVID‐19 patients. A recent study carried out on 5688 COVID‐19 patients [27] reports 334 patients with cancer (6% of the total), mostly with breast cancer, prostate cancer, lung cancer, urothelial cancer and colon cancer (57, 56, 23, 18 and 16 patients, respectively). COVID‐19 patients with cancer had more severe conditions (i.e. were intubated more frequently than non‐cancer patients, especially older patients; relative risk = 1.76). The investigation of a larger number of COVID‐19 patients is required to correlate the severity of clinical conditions with cancer type and cancer stage.

Here, we highlight the relevant association of TMPRSS2 with prostate cancer. TMPRSS2 is an androgen‐regulated gene that helps coronavirus entry into cells. Several studies propose that TMPRSS2 is a prostate cancer marker, as fused with the ERG gene. We then hypothesize that coronavirus infection, related at least in part to TMPRSS2 expression, might be associated with prostate cancer risk to some extent. Furthermore, the analyses carried out have revealed that different cancer types, as well as carcinogenesis and cancer metastasis, are associated with such five genes; in our opinion this may explain, at least in part, why cancer is reported as one of the main comorbidities in coronavirus infection [2, 28], namely hematological malignancies, colorectal cancer and lung cancer [29]. We speculate that the frequent cancer co‐occurrence in COVID‐19 patients may not be a casual or age‐related event; rather, it may associate with the specific expression patterns of SARS‐Cov‐2 receptors in the kidney, prostate, testis, thyroid, skin and other organs. Surprisingly, combining data from Fig. 3 and from Table 3 led us to observe that differential expression in cancers occurs mostly in body districts where these genes are highly expressed. Such correspondence is highlighted as red bars in Fig. 3: ACE2 is highly expressed in the gallbladder, testis, kidney and colon normal tissues (1st, 2nd, 3rd and 4th in rank) and consistently shows significant different expression in cholangiocarcinoma, testis, kidney and colon cancers (i.e. in the corresponding body districts). Similarly, TMPRSS2 shows the highest expression in colon, kidney and lung normal tissues (1st, 4th and 5th in rank) and consistently shows a significant different expression in colon, kidney and lung cancers. Similarly, CLEC4M shows high expression levels in liver and ovary normal tissues (1st and 4th in rank) and has differential expression in liver and ovary cancers. Finally, DDP4 is highly expressed in kidney (2nd in rank) and has significant differential expression in kidney cancer. We found relevant and significant changes in the expression of three genes, namely TMPRSS2, CLEC4M and DPP4. In more detail, a significant reduction of TMPRSS2 and CLEC4M in kidney and liver cancers, as well as a significant increase of DPP4 in thyroid cancer, was highlighted. The AUC was > 0.81, and so it is relevant to propose these as possible molecular markers for further investigation.

We propose the molecular basis explaining why COVID‐19 patients may have high risk of showing (at present or developing in the future) diseases such as diabetes, cardiovascular diseases and different cancer types. The COVID‐19 outbreak remains in progress at the time of the present study, and epidemiological studies that are carried out with respect to the medium‐ to long‐term follow‐up of such patients will confirm (or not) such a hypothesis. However, at this time, some clinical evidence is emerging. Kawasaki disease is a rare systemic vasculitis; its frequency in the past 3 months has demonstrated a 30‐fold increase in an Italian region showing a very high rate of COVID‐19 diffusion. According to a recent study [30] a significant association of Kawasaki disease with SARS‐CoV‐2 has been observed. Kawasaki disease has been proposed as being related to coronavirus infection, although this is still a debatable issue [31], whereas a genetic association of Kawasaki disease with ACE gene polymorphism has been confirmed [32] and the role of ACE2 in vasculitis and the control of endothelial wall physiology is known [33]. In addition, mice transgenic for human ACE2 show signs of vasculitis [34]. Furthermore, TMPRSS11D has been indicated as possible target gene of miRNAs, comprising biomarkers of Kawasaki disease [35], further linking SARS‐Cov‐2 receptors to Kawasaki disease.

One additional observation should be highlighted: gender and age are known to play a key role as risk‐ or protective‐factors in the most serious and lethal forms of COVID‐19 patients. Indeed, COVID‐19 epidemiology reveals that men and the elderly are largely more seriously affected than women and younger patients/children [36]. Noteworthy, the genes under investigation in the present study appear to be strongly related to the endocrine axis (disease class named ‘REPRODUCTION’ in Fig. 2) and to aging (disease class named ‘AGING’ in Fig. 2). Thus, we hypothesize that the functional connections of coronavirus receptors with prostate cancer and the ‘AGING’ and ‘REPRODUCTION’ disease classes may at least in part underlie the age and sex epidemiological features of COVID‐19 patients. As a final note, the disease class that best associates to the molecular network of the five coronavirus genes is ‘IMMUNE’ (Fig. 2). It is not surprising that molecules related to the virus entry associate with this class, although this finding underlies these molecules as potential triggers of both immune‐related viral infections and other diseases, such as endocrine‐related and cancer‐related diseases.

According to the results of the present study, we suggest the tissue expression of these coronaviruses receptors/interactors, as well as their association with specific diseases and differential expression in cancer types, may represent, at least in part, the molecular basis of COVID‐19 comorbidities. We propose that further investigation of these molecules may help controlling COVID‐19 comorbidities or may improve the follow‐up of patients who have recovered from this infection. The possible occurrence of still unrecognized comorbidities is also suggested.

A molecular approach somewhat comparable to the one proposed in the present study, although limited to ACE2 and TMPRSS2 receptors, has been published during the submission process of our study [37].

## Conclusions

According to the large tissues distribution of coronavirus receptors, as well as their association with different diseases and the highly significant differential expression in cancer types, we propose, for the first time, that coronavirus receptors are molecularly related to the most frequent COVID‐19 comorbidities, including cancers.

## Materials and methods

### Expression data in normal tissues

The expression level of ACE2, TMPRSS2, CLEC4M, DPP4 and TMPRSS11D in 32 human normal tissues was derived from the GENT2 database [38]. Transcriptomic data from this database are derived from the NCBI GEO database (https://www.ncbi.nlm.nih.gov/geo/) obtained by the Affymetrix U133A and U133Plus2 microarray platforms (Thermo Fisher Scientific, Waltham, MA, USA). Table S1 reports the number of healthy controls as well as the number of cancer patients investigated in the present study taken from the GENT2 database [38].

### Investigating functional relationship with body districts

chilibot analysis [39] measures the co‐occurrence of the chosen keywords in the same sentence, within PubMed‐indexed manuscripts, allowing to distinguish between interactive (stimulatory or inhibitory) and non‐interactive relationships. The tool named ‘relationships between two lists’ was used in the present study. The first list contained the word ‘coronavirus’; the second list contained the words depicting all body districts. The search was carried out on 22 March 2020; the ‘show only interactive relationships’ filter was activated. The strength of the interactive relationships was measured as function of number of PubMed‐indexed references supporting it. The default set‐up conditions were used, which stops when the analysis identifies 30 supporting abstracts.

### Investigating gene association with human diseases

Association of the five genes to human diseases was investigated exploiting different complementary approaches. The first analysis was carried out via the DisGeNET database, a large genes collection involved in human diseases; it allows the identification of genes associated with human diseases and their comorbidities [40]. An additional analysis was carried out using genemania (https://genemania.org) [41]. Each gene was singularly analyzed to obtain a list of 20 genes most related to it. genemania selects the related genes on the basis of protein–protein and protein–DNA interactions, common pathways, reactions, gene and protein expression data, protein domains, and phenotypic screening profiles, using publicly available databases. The five lists (each composed of 1 + 20 genes) were analyzed singularly and combined using DAVID Bioinformatics Resources, version 6.8 [42, 43], looking for gene‐annotation enrichment analysis of gene–diseases association. The Genetic Association Database [44]) was used to identify diseases and disease classes associated with each list of genes and their combination.

### Gene expression levels in cancer types

Gene expression levels of the five genes were investigated in two public cancer‐expression databases. Analyses were first carried out via the GEPIA2 database [45]. Boxplot analysis was carried out with a significance cut‐off of *P* < 0.0001 in cancers versus TCGA and GTEx normal samples. Validation was carried out via the GENT2 database (http://gent2.appex.kr/gent2) [38] with a significance threshold of *P* < 0.001. Data from about 49 000 healthy and cancer individuals were analyzed in more than 30 different cancer types. More details on the cancer types investigated and the number of patients and controls present in the GEPIA2 and GENT2 databases are provided in Table S1.

### Ethical statement

The data analyzed in the present study are all derived from anonymous public databases, with no ethical concerns.

### Statistical analysis

ROC analysis was carried out on the expression levels of the five genes in the cancer types and healthy controls, available from the GENT2 database. AUC was computed with ROC analysis, a commonly used method to perform binary classification. In this case, the two classes were ‘healthy controls’ on the one side, and ‘kidney cancer’ or ‘liver cancer’ or ‘thyroid cancer’ on the other. AUC measures the ability of the classifier to effectively distinguish the two classes, ranging from 0.5 (corresponding to 50% ability, i.e. by chance) to 1 (corresponding to 100% ability to distinguish healthy controls from cancer individuals). AUC was calculated using prism, version 6.01 (GraphPad Software Inc., La Jolla, CA, USA). *P* < 0.001 was considered significantly different, unless specified. Boxes and whiskers graphs were obtained using excel (Microsoft Corp., Redmond, WA, USA).

## Conflict of interests

The authors declare that they have no conflicts of interest.

## Author contributions

Antonio Facchiano, Francesco Facchiano and Angelo Facchiano contributed to study conceptualization, writing and analysis. Antonio Facchiano and Francesco Facchiano contributed to the methodology. Antonio Facchiano and Angelo Facchiano contributed to study validation. Antonio Facchiano contributed to funding acquisition.

## Supporting information


**Fig. S1.** Protein and RNA expression in different body districts. NX stands for ‘Normalized eXpression’, according to Human Protein Atlas (HPA) database.
**Table S1.** Cancer classification and individuals in each cancer category in the GEPIA2 and GENT2 databases. Cancer types are classified with different names in the two databases. For example, GEPIA2 classifies three different kidney cancer types, whereas GENT2 indicates only one general kidney cancer classification. Also, GEPIA2 has two distinct classifications for colon and rectum carcinomas, whereas GENT2 has one generic colon cancer group. The paired classifications indicated were used to validate GEPIA2 data in the GENT2 database.
**Table S2.** DAVID/Genetic Association Database (GAD) analysis for the five genes with related genes obtained by genemania analysis. The list of GAD diseases associated with each of the five genes is reported below. In the case of ACE2, the list has been restricted to the top 25 diseases.

## Data Availability

Data and details of the analyses are available from the corresponding author upon reasonable request.
