# Supplementary material for: An investigation into the molecular basis of cancer comorbidities in coronavirus infection
Source: FEBS Open Bio. 2020 Oct 12;10(11):2363–74. doi: 10.1002/2211-5463.12984 (PMC7537529; doi:10.1002/2211-5463.12984)
Supplement: Supplementary file 1 — Fig. S1. Protein and RNA expression in different body districts. NX stands for ‘Normalized eXpression’, according to Human Protein Atlas (HPA) database. Table S1. Cancer classification and individuals in each cancer category in the GEPIA2 and GENT2 databases. Cancer types are classified with different names in the two databases. For example, GEPIA2 classifies three different kidney cancer types, whereas GENT2 indicates only one general kidney cancer classification. Also, GEPIA2 has two distinct classifications for colon and rectum carcinomas, whereas GENT2 has one generic colon cancer group. The paired classifications indicated were used to validate GEPIA2 data in the GENT2 database. Table S2. DAVID/Genetic Association Database (GAD) analysis for the five genes with related genes obtained by genemania analysis. The list of GAD diseases associated with each of the five genes is reported below. In the case of ACE2, the list has been restricted to the top 25 diseases. [file FEB4-10-2363-s001.pdf]

An investigation into the molecular basis of cancer comorbidities in coronavirus infection

Antonio Facchiano[1*#], Francesco Facchiano[2#], Angelo Facchiano[3#]

[1] Istituto Dermopatico dell'Immacolata, IDI-IRCCS, Rome, Italy;
email: a.facchiano@idi.it

[(2)] Department of Oncology and Molecular Medicine, Istituto Superiore di Sanità,
Rome, Italy; email: francesco.facchiano@iss.it

[(3)] Institute of Food Science, National Research Council, Avellino, Italy;
email: angelo.facchiano@isa.cnr.it

* Correspondence: Antonio Facchiano, a.facchiano@idi.it

SUPPLEMENTARY MATERIAL

Figure S1. Protein and RNA expression in different body districts. NX stands for "Normalized eXpression", according to Human Protein Atlas (HPA) database

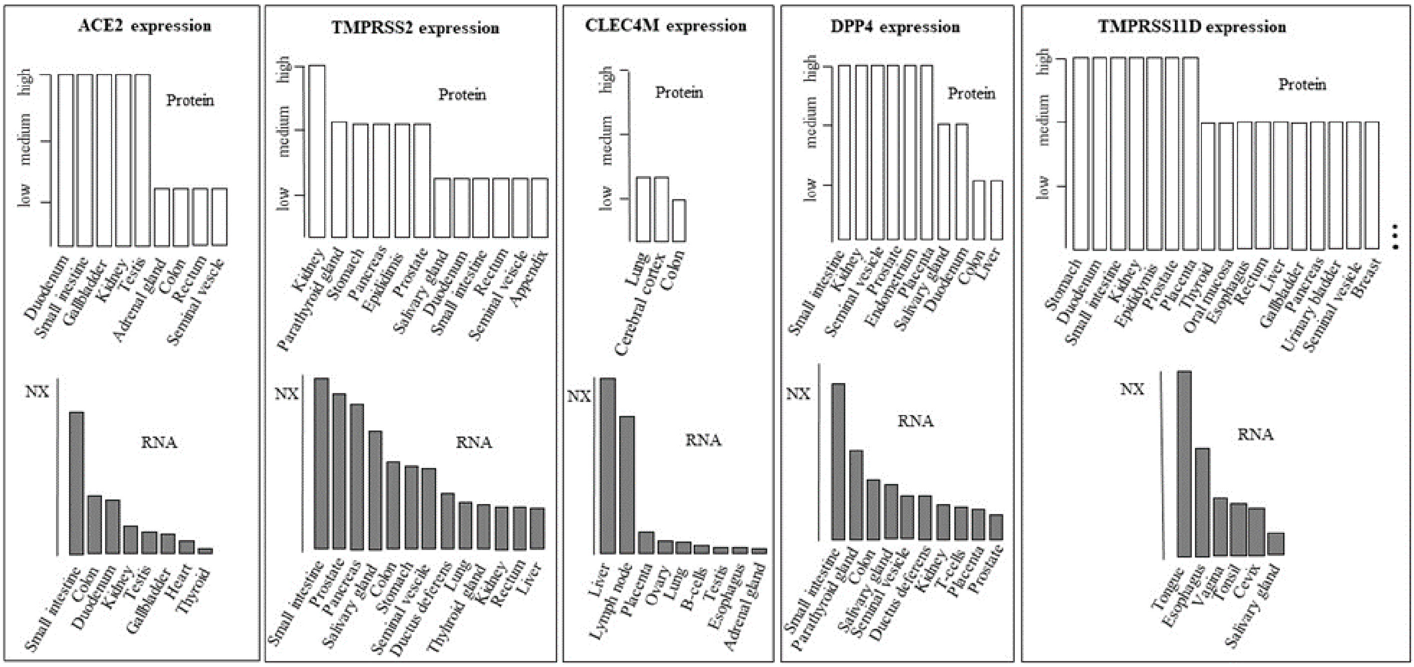

Supplementary Table 1. **Cancer classification and individuals in each cancer category in GEPIA2 and GENT2 databases.** Cancer types are classified with different names in the two databases. For instance, GEPIA2 classifies 3 different kidney cancer types, while GENT2 indicates only 1 general kidney cancer classification. Also, GEPIA2 has two distinct classifications for colon and rectum carcinomas while GENT2 has one generic colon cancer group. The paired classifications as indicated in the table were used to validate GEPIA2 data in GENT2 database.

**GEPIA database**			**GENT2 database**			**Totals**
	pz	ctrl		pz	ctrl	
Adrenocortical carcinoma	77	128	Adrenal gland cancer	113	18	**336**
Bladder Urothelial Carcinoma	404	28	Bladder cancer	191	58	**681**
Breast invasive carcinoma	1085	291	Breast cancer	6190	484	**8050**
Cervical squam. cell carcioma Endocervical adenocarcinoma	306	13	Cervix cancer	117	10	**446**
Cholangio carcinoma	36	9				**45**
Colon adenocarcinoma	275	349	Colon cancer	4046	398	**5068**
Lymph neopl diff large B-cell lymph	47	337	Lymph node cancer	55	1	**440**
Esophageal carcinoma	182	286	Esophagus cancer	269	23	**760**
Glioblastoma multiforme	163	207				**370**
Head & Neck squam cell carc	519	44	Head and Neck cancer	216	19	**798**
Kidney chromophobe	66	53	Kidney cancer	874	289	**2251**
Kidney renal clear cell carc.	523	100				
Kidney renal papill. cell carc.	286	60				
Acute Myeloid Leukemia	173	70				**243**
Brain Lower Grade Glioma	518	207	Brain Cancer	3552	872	**5149**
Liver hepatocell carcarc	369	160	Liver cancer	538	214	**1281**
Lung adenocarcinoma	483	347	Lung cancer	2648	507	**4709**
Lung squam. cell carcinoma	486	238				
Ovarian serous cystadenocarc	426	88	Ovary cancer	1651	109	**2274**
Pancreatic adenocarcinoma	179	171	Pancreas cancer	355	104	**809**
Pheochromocyt and paraganglioma	12	3				**15**
Prostate adenocarcinoma	492	152	Prostate cancer	331	55	**1030**
Rectum adenocarcinoma	92	318				**410**
Sarcoma	262	2				**264**
Skin cutaneous melanoma	461	558	Skin cancer	754	288	**2061**
Stomach adenocarcinoma	408	211	Stomach cancer	1112	116	**1847**
Testicular Germ Cell Tum.	137	165	Testis cancer	4	8	**314**
Thyroid carcinoma	512	337	Thyroid cancer	325	200	**1374**
Thymoma	118	339				**457**
Uterine corpus endometr carc.	174	91	Endometrium cancer	473	72	**810**
Uterine carcinosarcoma	57	78	Uterus cancer	512	57	**704**
**Totals**	**9328**	**5440**		**24326**	**3902**	**42996**

Supplementary Table 2.

DAVID / Genetic Association Database (GAD) analysis for the 5 genes with related genes obtained by Genemania analysis.

The list of GAD diseases associated to each of the 5 genes is reported below. In the ACE2 case the list has been restricted to the top 25 diseases.

Gene	GAD_DISEASE Term	Count	%	P Value	Genes
	nephropathy, diabetic	4	20	6.69E-06	ACE, AGT, ACE2, GHRL
	sudden cardiac arrest risk	3	15	3.48E-05	ACE, AGT, ACE2
	Atrial Fibrillation	3	15	9.67E-05	ACE, AGT, ACE2
	Pregnancy Complications	3	15	1.48E-04	ACE, AGT, CAT
	Chronic renal failure|Kidney Failure, Chronic	7	35	3.28E-04	SLC12A6, FRK, ACE, AGT, GHRL, CAT, SLC10A2
	left ventricular hypertrophy	3	15	5.33E-04	ACE, AGT, ACE2
	Type 2 diabetes	5	25	6.25E-04	ACE, AGT, ACE2, GHRL, CAT
	Insulin Resistance	4	20	7.93E-04	ACE, AGT, GHRL, SLC10A2
	DNA damage	3	15	9.97E-04	ACE, AGT, CAT
	blood pressure, arterial	3	15	0.001854	ACE, AGT, ACE2
	Chronic renal failure|Diabetic Nephropathies|Diabetic Nephropathy|Hypertension|Kidney Failure, Chronic	2	10	0.0023	ACE, AGT
	Brain Ischemia|Carotid Artery Diseases	2	10	0.0023	ACE, AGT
	Nonfamilial Hypertrophic or Dialated Cardiomyopathy	2	10	0.0023	ACE, AGT
	diabetes, type 2; polymetabolic syndrome	2	10	0.0023	ACE, AGT
	Marfan Syndrome|Mitral Valve Prolapse	2	10	0.0023	ACE, AGT
	obesity; blood pressure, arterial; sleep apnea	2	10	0.0023	ACE, AGT
	Adult Respiratory Distress Syndrome|Chromosome Deletion|Respiratory Distress Syndrome, Adult	2	10	0.0023	ACE, AGT
ACE2	Chronic renal failure|Hypertension|Kidney Failure, Chronic	2	10	0.0023	ACE, AGT
	Hypertension|Polycystic Kidney Diseases	2	10	0.0023	ACE, AGT
	Kidney Failure|kidney; failure|Lupus Nephritis|Nephritis SLE	2	10	0.0023	ACE, AGT
	Brain Infarction|Brain Ischemia|Cerebrovascular Disorders|Hypertension|Leukoaraiosis	2	10	0.0023	ACE, AGT

	Albuminuria|Diabetes Mellitus, Type 2|Diabetic Nephropathies	2	10	0.0023	ACE, AGT
	hypertension; renal dysfunction, posttransplantation	2	10	0.0034	ACE, AGT
	Fibromuscular Dysplasia|Hypertension|Renal Artery Obstruction	2	10	0.0034	ACE, AGT
	Chronic renal failure|Kidney Failure, Chronic|Uremia|Vesico-Ureteral Reflux|Vesicoureteral reflux	2	10	0.0034	ACE, AGT
TMPRSS2	prostate specific antigen	2	10	0.0027	AR, KLK3
	clinical parameters and endocrine status in elderly men	2	10	0.0041	AR, KLK3
	prostate cancer	5	24	0.0046	TMPRSS2, AR, KLK2, KLK3, KLK4
	prostate volume/histology endocrine patterns	2	10	0.0069	AR, KLK3
	Depression	3	14	0.0193	AR, EMX2, PDE9A
	prostatic hyperplasia	2	10	0.0301	AR, KLK3
	Azoospermia|Oligospermia	2	10	0.0659	AR, KDM3A
	Personality	2	10	0.0762	AR, NGF
CLEC4M	Severe Acute Respiratory Syndrome	3	14	5.50E-04	CLEC4G, CD209, CLEC4M
	HIV Infections|Sexually Transmitted Diseases	2	10	0.0043	CD209, CLEC4M
	HIV Infections	3	14	0.0044	CD209, TLR2, CLEC4M
	Leprosy	3	14	0.0052	FCN2, CD209, TLR2
	Vaginosis, Bacterial	2	10	0.0087	CRHBP, TLR2
	Glomerulonephritis, IGA	3	14	0.0120	TLR2, CETP, ITGAM
	Pregnancy Complications, Infectious|Vaginosis, Bacterial	2	10	0.0131	TLR2, LBP
	chronic obstructive pulmonary disease	4	19	0.0213	TLR2, TFF3, CD4, CETP
	Malaria, Cerebral	2	10	0.0217	TLR2, TIMD4
	Cardiovascular Diseases	3	14	0.0306	SAA1, TLR2, CETP
	Birth Weight|Leukemia|Leukemia, Myeloid, Acute|Precursor Cell Lymphoblastic Leukemia-Lymphoma	3	14	0.0362	CD209, TLR2, LBP
	HIV	3	14	0.0365	CD209, TLR2, CLEC4M
	Meningeal Neoplasms|meningioma	3	14	0.0372	CD209, TLR2, LBP
	lung cancer	4	19	0.0377	TLR2, TFF3, CD4, CETP
	Bladder Cancer	4	19	0.0401	TLR2, TFF3, CD4, CETP
DPP4	Bulimia	5	24	1.26E-04	GRP, PREP, MAOA, IGF1, DPP4
	Type 2 Diabetes| edema | rosiglitazone	9	43	0.0086	BCKDK, MAOA, DPP10, IGF1, DPP6, CXCL12, DPP4, ADA, FN1
	Lymphopenia|SCID|Severe Combined Immunodeficiency	2	10	0.0131	PTPRC, ADA

	anxiety disorder	2	10	0.0188	MAOA, CAMKK2
	Bipolar Disorder	4	19	0.0204	PREP, MAOA, DPP10, CAMKK2
	diabetes, type 2	4	19	0.0249	PREP, PDK4, IGF1, ADA
	diabetes, type 1	3	14	0.0309	PTPRC, CXCL12, ADA
	systemic sclerosis	2	10	0.0331	PTPRC, FN1
	mental retardation	2	10	0.0331	MAOA, ADA
	Coronary Artery Disease	4	19	0.0366	PREP, IGF1, CXCL12, FN1
	Scoliosis	2	10	0.0374	IGF1, DPP9
	Colonic Neoplasms	2	10	0.0528	IGF1, ADA
	Lung Cancer	4	19	0.0534	MAOA, IGF1, CXCL12, FN1
	Birth Weight	2	10	0.0598	IGF1, ADA
	several psychiatric disorders	3	14	0.0700	GRP, MAOA, ADA
	panic disorder	2	10	0.0735	GRP, MAOA
	autism	3	14	0.0950	MAOA, IGF1, ADA
TMPRSS11D	Eczema	2	10	0.0099	KLK7, SPINK5
	Dermatitis, Atopic|Eczema allergic	2	10	0.0237	KLK7, SPINK5
	Dermatitis, Atopic|	2	10	0.0374	S100A7, SPRR1B
	prostate cancer	3	14	0.0904	KLK7, KLK12, KLK13
